# Heat Risk Perception and Vulnerability in Puerto Rico: Insights for Climate Adaptation in the Caribbean

**DOI:** 10.3390/ijerph22081197

**Published:** 2025-07-31

**Authors:** Brenda Guzman-Colon, Zack Guido, Claudia P. Amaya-Ardila, Laura T. Cabrera-Rivera, Pablo A. Méndez-Lázaro

**Affiliations:** 1 Department of Environmental Health, Graduate School of Public Health, University of Puerto Rico-Medical Sciences Campus, San Juan 00936-5067, Puerto Rico; brenda.guzman3@upr.edu (B.G.-C.); laura.cabrera@upr.edu (L.T.C.-R.); 2 Arizona Institute for Resilience, University of Arizona, Tucson, AZ 85721, USA; zguido@arizona.edu; 3 Department of Biostatistics, Graduate School of Public Health, University of Puerto Rico-Medical Sciences Campus, San Juan 00936-5067, Puerto Rico; claudia.amaya@upr.edu; 4 Cancer Biology Division and Environmental Translational Cancer Program, Comprehensive Cancer Center, San Juan 00936-5067, Puerto Rico

**Keywords:** heat risk perception, social vulnerability, climate adaptation, community resilience, Caribbean

## Abstract

Extreme heat poses growing health risks in tropical regions, yet public perception of this threat remains understudied in the Caribbean. This study examines how residents in Puerto Rico perceived heat-related health risks and how these perceptions relate to vulnerability and protective behaviors during the extreme heat events of the summer of 2020. We conducted a cross-sectional telephone survey of 500 adults across metropolitan and non-metropolitan areas of Puerto Rico, using stratified probability sampling. The questionnaire assessed heat risk perception, sociodemographic characteristics, health status, prior heat exposure, and heat-related behaviors. While most participants expressed concern about climate change and high temperatures, fewer than half perceived heat as a high level of personal health risk. Higher levels of risk perception were significantly associated with being male, aged 50–64, unemployed, and in fair health, having multiple chronic conditions, and prior experience with heat-related symptoms. Those with symptoms were nearly five times more likely to report high levels of risk perception (OR = 4.94, 95% CI: 2.93–8.34). In contrast, older adults (65+), despite their higher level of vulnerability, reported lower levels of risk perception and fewer symptoms. Nighttime heat exposure was widespread and strongly associated with heat-related symptoms. Common coping strategies included the use of fans and air conditioning, though economic constraints and infrastructure instability limited access. The findings highlight the disparity between actual and perceived vulnerability, particularly among older adults. Public health strategies should focus on risk communication tailored to vulnerable groups and address barriers to heat adaptation. Strengthening heat resilience in Puerto Rico requires improved infrastructure, equitable access to cooling, and targeted outreach.

## 1. Introduction

Exposure to elevated temperatures can lead to heat-related illnesses, exacerbate existing health conditions, negatively impact worker productivity, and hinder physical activity capacity. Extreme heat poses significant health risks, including increased mortality and morbidity, especially in cardiovascular and respiratory diseases [1,2,3,4,5,6,7,8].

Global evidence indicates increased vulnerability and exposure of populations to heatwaves [9]. While elevated temperatures affect all individuals, certain population groups exhibit increased vulnerability due to specific risk factors and social determinants of health. Groups at increased risk of heat-related illness or death include adults over 65 years of age, children, people with pre-existing medical conditions, outdoor workers, people living in poverty, and those who are socially isolated [10]. It has also been documented that residents of urban areas exhibit higher risks of heat exposure and health consequences owing to the heat island effect. Social determinants of health, such as income, educational level, social cohesion, housing and living conditions, and minority group status, also influence health risks associated with extreme heat events [8,9,11,12]. These factors often interact, increasing the risk among people with multiple vulnerabilities.

Heat risk perception refers to how people understand and determine their personal risks related to their exposure to high temperatures and their potential impact on health. Heat risk perception varies across populations and is influenced by structural, environmental, personal, and social factors. These factors influence how individuals understand their risk and whether they adopt protective measures to minimize exposure [11]. Outdoor environmental factors include meteorology (e.g., wind speed and direction, sun radiation, temperature, water vapor, and relative humidity) and climate (summer, fall, winter, and spring). Personal factors include thermal comfort, adaptive measures, and health concerns, whereas structural factors include communication systems, barriers to adaptation, and availability of resources [11]. The social factors include sociodemographic aspects and social support. Socioeconomic status, educational attainment, and health status are significant determinants of risk perception, with those with low income, higher education, and health problems generally having a higher level of risk perception [11,13,14,15,16,17,18]. Demographic factors, such as age, gender, and ethnicity, also impact perception, although these may vary by culture and context [4,13,18,19]. Occupation, especially for those who work outdoors, may increase risk perception [4,11,16,17]. Additionally, social cohesion and previous experiences with heat-related problems tended to increase awareness and perception of risk [11].

Although studies have been conducted on heatwave risk perception and adaptive behaviors, there are still significant gaps in knowledge on this topic, especially regarding the social and cultural factors that influence how people perceive and respond to extreme heat risks. There are also a few studies on heat risk perception in specific demographic and geographic contexts, with most studies concentrated in the United States, China, and Australia [11]. There is a need to expand our knowledge on this topic, especially in vulnerable populations, rural areas, and other regions, to formulate context-specific mitigation and adaptation strategies.

In the Caribbean, research on climate extremes and health implications is limited, with few studies addressing public health readiness and responses to severe weather, food and water security, the direct impact of increasing temperatures, and how the public perceives these effects [20]. In Puerto Rico, while there is a growing interest in climate perception, there remains a significant gap in research specifically focused on the public’s perception of thermal or heat-related risk. This lack of evidence is particularly concerning, given the island’s high vulnerability to extreme heat events and their potential health consequences. Addressing this gap is essential for developing context-specific policies and public health interventions. Understanding how individuals perceive heat-related risks can enhance the effectiveness of heat alert systems, guide resource allocation for cooling infrastructure, and support targeted communication strategies to protect vulnerable populations. Recent studies have suggested that the public is increasingly concerned about climate change, especially in relation to rising temperatures [21,22,23], reinforcing the urgency of advancing research linking heat risk perception to actionable policy responses in Puerto Rico and similar settings across the Caribbean.

Temperatures in Puerto Rico and the Virgin Islands have increased by nearly 2 °F since 1950, with projected warming of up to 7 °F above the historical record in a higher-emission scenario [24]. Extreme heat conditions in the Caribbean have intensified since 1980, with increasing trends in the number of heat-stressed days (>39.9 °C (103.8 °F)) [25]. Since 2000, the number of extremely hot nights has generally been above average, with higher numbers being recorded since 2015 [24]. The urban heat island effect caused temperatures in San Juan to rise faster than those on the rest of the islands [24]. Climatological analyses have identified the August–September–October period as the season with the highest levels of heat stress in the Caribbean. The year 2020 was one of the three warmest years in the region and set temperature records in Cuba, Puerto Rico, Dominica, and Grenada, reflecting an ongoing warming trend since the 1990s [25]. This year, two heatwaves were documented in April and September, which affected the region and exposed the population to unprecedented heat conditions [25].

In a warmer scenario, there are limited studies on climate perception in Puerto Rico. There is a notable absence of research that specifically addresses heat-related health risk perceptions. This gap underscores the need for targeted investigations to enhance our understanding of how climate-related risks impact community health and well-being and to inform the development of effective mitigation policies and programs.

This study aimed to examine the perception of risk, vulnerability, attitudes, and knowledge concerning heat in Puerto Rico during the summer of 2020. This research aims to determine whether individuals with greater vulnerability—due to age, chronic health conditions, unemployment, or lower educational attainment—report higher levels of perceived risks and greater concerns about heat-related health impacts. This analysis was guided by the Health Belief Model (HBM), a widely recognized theoretical framework for understanding how individuals perceive health risks and make decisions to adopt protective behaviors. According to the HBM, individuals are more inclined to take action if they perceive themselves as being susceptible to a health threat, believe that the threat has serious consequences, and are convinced that specific actions can mitigate it [26]. In the context of extreme heat, it is anticipated that individuals′ perception of personal risk, prior experiences with heat-related symptoms, and awareness of available resources will influence both their level of concern and protective behaviors. We hypothesized that individuals with a poor health status, previous experience with heat-related symptoms, or markers of social vulnerability are more likely to perceive high levels of heat-related health risks.

## 2. Materials and Methods

### 2.1. Study Area

**Figure 1:**
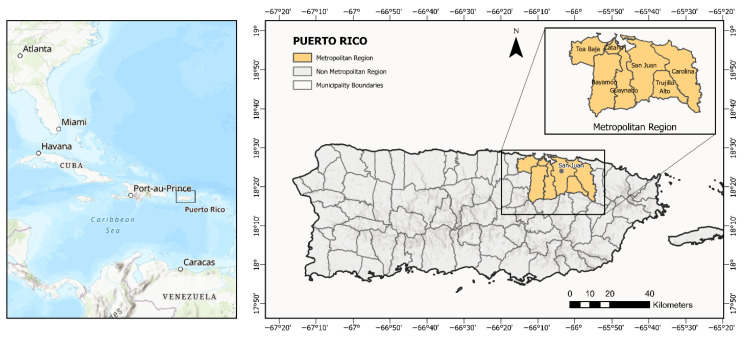
Location of Puerto Rico in the Caribbean and delineation of study regions by municipality.

Puerto Rico is an island located in the north-central Caribbean Sea (17.92° N–18.52° N, 65.62° W–67.28° W) with a tropical climate (Figure 1). The territory is divided into 78 municipalities, with a total population of approximately 3,285,874 inhabitants. The geographic scope of this study encompasses Puerto Rico. For the purposes of sample stratification and comparative analysis, two regions were delineated: the metropolitan region, which includes the municipalities of Bayamón, Carolina, Cataño, Guaynabo, San Juan, Toa Baja, and Trujillo Alto, and the non-metropolitan region, which consists of the remaining municipalities of Puerto Rico.

Extreme weather events in Puerto Rico include tropical cyclones and rising temperatures, with increasing frequencies of extreme heat and reduced rainfall [27]. Summers are characterized by consistent easterly trade winds and relatively stable temperatures, with daily highs ranging from 25 to 35 °C (77–95 °F) and lows between 20 and 25 °C (68–77 °F) [28]. In the San Juan metropolitan region, hot days often bring sunny skies and higher temperatures along the northern coast [28].

### 2.2. Survey Methods

This cross-sectional study used telephone surveys to collect data from a probabilistic sample of 500 adults aged 21 and older who resided in Puerto Rico during the summer of 2020 (June–September). The survey was co-designed by teams from the University of Arizona and the University of Puerto Rico Medical Sciences Campus. Participants were selected using proportionally stratified probability sampling based on geographic region (metropolitan vs. non-metropolitan) and demographic characteristics according to the 2018 Puerto Rico Community Survey (5-year estimate) (Table 1). Random digit dialing (RDD), following the Mitofsky–Waksberg method [29], was used to select telephone numbers. Estudios Tecnicos, Inc. conducted the interviews between 23 January and 22 March 2021. A total of 46,260 calls were made. The maximum sampling error was ±4.4% at a 95% confidence level.

The 40-question survey covered demographics, socioeconomic status, heat exposure, risk perception, information and institutional trust, heat-related behaviors, perceived exposure, health, and compound climate hazards, using predefined responses, open-ended questions, hybrid formats, and Likert scales. Informed consent was obtained from all the participants, and the data were processed using SPSS and Excel to ensure consistency, confidentiality, and anonymity. The study was approved by the University of Arizona IRB (protocol: 1812204202A003).

### 2.3. Data Analysis

Statistical analyses included variables on sociodemographic characteristics (region, age, gender, education, employment, and household size), health status, heat exposure, behaviors, and risk perception. Due to a 55% non-response rate, household income was excluded; however, education and employment were included as proxies for socioeconomic status. The health variables encompassed the self-perceived health status, which was selected from a range of five levels, from excellent to very poor. Additionally, health conditions and health insurance were considered. The inquiries addressed aspects such as thermal comfort, experiences with heat-related symptoms during the summer months, sleeping at uncomfortable temperatures, actions taken to stay cool at home, indicators of air conditioning (AC) usage, and the availability of alternative energy systems within the residential context. A full description of all the variables and their coding is provided in Appendix A Table A1.

The assessment of the heat risk perception entailed inquiries concerning the degree of concern regarding health in response to elevated temperatures and the probability of encountering a heat-related illness necessitating medical intervention within a five-year timeframe at the individual, family, and community levels. This approach enabled the independent evaluation of various dimensions of heat-related health risk perception. Risk perception was assessed using five-point Likert scales, later recoded as low, moderate, and high levels; immediate concern about high temperatures served as the primary indicator. In contrast, perceived future risk, as assessed in this study, pertains to the anticipated concern of developing illnesses requiring medical attention in the medium term. This suggests the necessity of evaluating future risk and vulnerability, as well as the severity of health impacts from heat events. These distinctions are significant because they may correspond to varying levels of action and adaptive behaviors. Furthermore, the survey included an open-ended question regarding the primary concerns associated with elevated temperatures, which were categorized as direct and indirect health effects.

Descriptive statistics and frequency distributions were used to analyze the study variables. To facilitate these analyses, certain variables were recoded into broad categories. Chi-squared tests were used to evaluate the associations between the variables of interest (region, sociodemographic, health status, perceived exposure, and heat-response behaviors) and the levels of risk perception. Additionally, chi-squared tests were used to evaluate the associations between the experience of heat-related symptoms and the variables of interest. We employed multinomial regression models to compare various categories of risk perception (low, moderate, and high levels) with potential predictor variables, utilizing the low-risk category as the reference point. Although the models estimated outcomes for both moderate and high levels of perceived risks, only the results for the high-risk category were presented, as they were more consistently significant and relevant to the study objectives. Initially, univariate analyses were conducted with each independent variable, followed by multivariate analyses to identify adjusted associations with risk perception. We used binary logistic regression analyses to evaluate the strengths of the associations between the predictor variables and prior experience with heat-associated symptoms (dichotomized as yes/no). Initially, univariate analyses were performed, followed by multivariate models adjusted for multiple independent variables. A significance level of 5% (*p* < 0.05) was used. Frequency and chi-squared analyses were conducted using SPSS Statistics (version 30), while logistic regression analyses were performed using STATA (version 18.5).

## 3. Results

### 3.1. Sociodemographic Characteristics of the Participants

The sample consisted of 500 adults across Puerto Rico, with the majority (72%) residing in non-metropolitan areas (Table 2). The mean age was 50.6 years, and 53% of the respondents were male. Most participants (61%) had completed post-secondary education, and 42% were employed at the time of the survey. Nearly one in five lived alone (Table 3), and 95% reported having health insurance coverage. A majority (69%) reported being in excellent or good health, while 71% had at least one chronic condition (Table 4). The most commonly reported conditions were cardiovascular (40%), neurological or sleep disorders (38%), and metabolic conditions (30%). Reports of fair or poor/very poor health were more prevalent among older adults, with 42.7% of the individuals aged 50 to 64 and 40.4% of those aged 65 and older indicating this health status.

### 3.2. Reported Thermal Comfort Threshold

**Figure 2:**
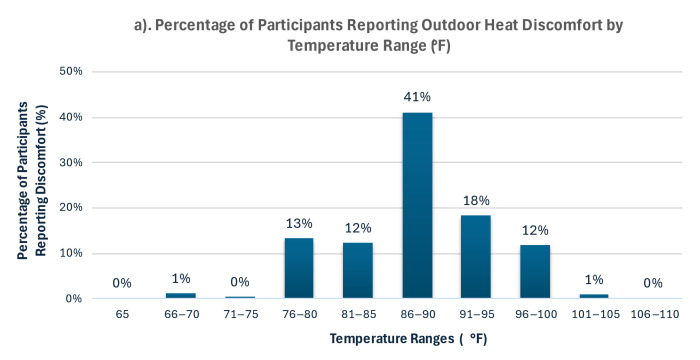

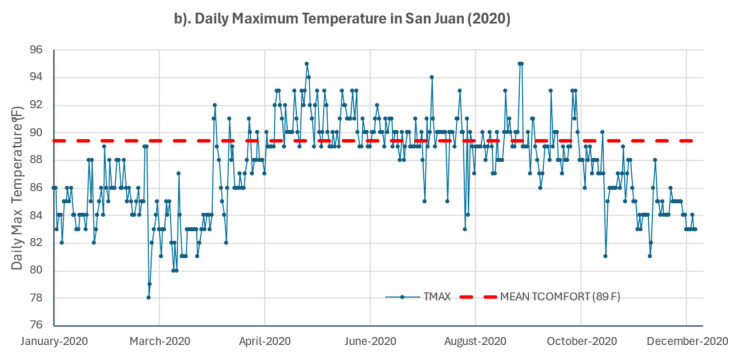
Outdoor heat discomfort and observed temperatures in San Juan, Puerto Rico. (**a**) Percentage of participants reporting the onset of outdoor heat discomfort by temperature range (F), based on survey responses. Percentages represent the first temperature range at which discomfort was perceived and are not cumulative. (**b**) Daily maximum temperature in San Juan (LM Marin International Airport, Station RQW00011641) for the year 2020, and mean thermal comfort threshold (89 °F) reported by survey participants (red dashed line).

Most participants (72%) reported feeling discomfort during outdoor activities at temperatures above 86 °F, with a mean thermal discomfort threshold of 89.4 °F (95% CI: 88.8–90.0) (Figure 2). This aligns with thermal comfort literature for tropical climates [30,31]. In 2020, this threshold was exceeded on 111 days, including 73 days (60%) during the summer months of June through September (Figure 2).

### 3.3. Health Risk Perception

Nearly half the participants (47%) reported a high level of concern about heat-related health risks, while fewer perceived themselves or their families as being likely to experience heat-related illness within five years (Table 5). Young adults (21–34) and older adults (65+) most frequently reported low levels of perceived risk. In contrast, concerns about climate change, high temperatures, and heatwaves were widespread, with over three-quarters of the respondents expressing high levels of concern.

Chi-squared tests showed that risk perception was significantly associated with region, age, gender, and employment status (Table 6). Adults aged 50–64 and those unable to work reported the highest levels of perceived risk, while those aged 65+ reported the lowest. Health risk perception was also higher among individuals in fair or poor health, those with multiple chronic conditions, and those who had previously experienced heat-related symptoms or uncomfortable sleep temperatures. No significant associations were found between perceived health risk and specific household heat mitigation behaviors, such as the use of air conditioning or visiting public spaces to cool down.

### 3.4. Future Health Risk Perception

Future health risk perception was assessed at the individual, family, and community levels over a five-year outlook. Chi-squared analyses revealed higher levels of perceived risk among men, adults aged 35–64, individuals with fair or poor health, those unemployed or unable to work, and respondents with lower educational attainment or multiple chronic conditions. Prior experience with heat-related symptoms and sleeping at uncomfortable temperatures were strongly associated with higher levels of perceived future risk across all the levels. Visiting public spaces to cool off was also linked to increased perceived risk at the family and community levels (Table 6).

### 3.5. Self-Reported Concerns About High Temperatures

**Figure 3:**
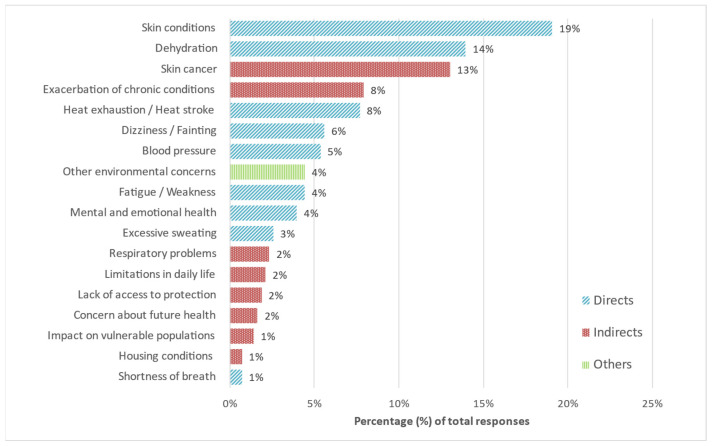
Reported concerns related to high temperatures.

Participants reported 430 concerns about high temperatures, which were categorized as direct health effects (59%), indirect effects (35%), and other environmental concerns (4%) (Figure 3). Direct concerns included dehydration, dizziness, and excessive perspiration. Indirect concerns involved the exacerbation of pre-existing conditions and limitations on daily activities. Despite well-established links between heat and cardiovascular or respiratory illness, these outcomes were rarely mentioned. Only 2% explicitly identified respiratory issues, and 8% referred to chronic conditions more generally. This gap highlights a disconnect between scientific evidence and public perception, underlining the need for risk communication strategies that raise awareness of severe but less visible heat-related health risks.

### 3.6. Perceived Heat Exposure

Forty-four percent (44%) of the respondents reported experiencing heat-related symptoms during the summer of 2020 (June through September) (Table 6). Among those who reported symptoms, 45% experienced them one to two times, 34% experienced them two to four times, and 22% experienced them five or more times during summer. The occurrence of heat-associated symptoms was most frequently reported among residents of non-metropolitan areas, men, individuals aged 50 to 64 years, households comprising two to three persons, individuals who were unemployed or unable to work, persons with lower educational levels, those who worked both indoors and outdoors, those who reported a fair health status, those who suffered from at least one chronic health condition, and those reporting thermal discomfort at temperatures ≤ 85 °F. Chi-squared tests indicated statistically significant associations between experiencing symptoms and gender (*p* = 0.023), educational level (*p* = 0.034), employment status (*p* = 0.01), health status (*p* < 0.001), and the number of health conditions (*p* < 0.001).

Sixty-eight percent (68%) reported having slept at uncomfortable temperatures; however, no statistically significant differences were observed by region or other sociodemographic variables, except for household size, which was more frequently reported among individuals living alone (*p* = 0.005).

### 3.7. Heat-Related Behaviors

About 45% of the participants reported visiting public places to cool down during the summer of 2020, though most did so infrequently. This behavior was more common among younger adults (21–34 years) and less frequent among older adults (65+ years), individuals unable to work, and those with lower educational attainment. To mitigate heat at home, the most common strategies were the use of fans (84%), air conditioning (64%), and natural ventilation. AC use was more prevalent in metropolitan areas and varied by duration, with nearly half using it 13 h or more per day. However, financial limitations were common; 43% reduced AC use due to cost, and 13% were unable to pay their electricity bill. About 61% of the respondents reported having backup power systems (Table 7). These behavioral patterns varied by demographic and health status, as shown in Table 8, highlighting that the most heat-vulnerable populations may also face structural and economic barriers to adaptation.

### 3.8. Predictors of Risk Perception and Heat Exposure

Multivariate logistic regression identified several factors significantly associated with high levels of heat risk perception, including non-metropolitan residence, male sex, age 50–64 years, and prior heat symptom experience (Table 9). Future risk perception at individual, family, and community levels showed distinct patterns of association with demographic factors and heat exposure experiences. Additionally, individuals with fair health, pre-existing chronic conditions, and those experiencing heat-related symptoms and uncomfortable sleeping temperatures showed increased likelihoods of reporting heat-related symptoms (Table 10).

## 4. Discussion

This study examined heat risk perception among 500 Puerto Rico residents during the record-breaking summer of 2020, when the Caribbean experienced unprecedented temperatures with significant heatwaves. While most participants expressed concern about climate threats, less than half perceived high levels of health risks from heat, and even fewer anticipated future heat-related illness.

Contrary to prior research suggesting women are more concerned about environmental threats, the men in this study reported higher levels of heat-related health risk perception. This may reflect differences in occupational exposure, cultural norms, or lived experiences. Although the work setting was not statistically significant, men may still encounter greater heat exposure. These findings highlight the importance of considering local gender roles and social context when designing public health interventions.

Statistically significant differences were observed in the levels of heat risk perception and perceived heat exposure between the two regions evaluated, with higher levels reported among the residents of the non-metropolitan area compared to the residents of the San Juan metropolitan area (an area of higher urban density), where greater exposure is presumed to be due to heat island effects. The observed differences could be related to possible acclimatization effects among the residents of the metropolitan area and to greater perceived vulnerability among the residents of the non-metropolitan area due to less access to heat mitigation resources. However, the study could not distinguish among rural, suburban, and urban zones, limiting the granularity of the geographic vulnerability analysis.

Older adults (65 years and older) reported the lowest levels of perceived health risks from heat exposure and the least experience of heat-associated symptoms, indicating a significant underestimation of their vulnerability. This finding aligns with previous studies, which have demonstrated that vulnerable groups, particularly older adults, often do not perceive a high level of risk and tend to underestimate their susceptibility to extreme heat [32,33]. Given their heightened vulnerability and low levels of risk perception, older adults should be considered as a critical target group for public health interventions aimed at increasing awareness, improving risk communication, and promoting protective behaviors during extreme heat events.

The participants with fair health and pre-existing chronic conditions reported higher levels of heat-related health risk perception. Risk perception increased progressively with the number of health conditions, particularly among those with two or more. These same factors were also associated with greater odds of experiencing heat-related symptoms. These findings are consistent with previous studies identifying poor health status and chronic illness as key drivers of vulnerability and perceived heat risk [11,13,14,15,16,17,18].

This study identified prior exposure to heat as a significant factor that influences health risk perception. Individuals who have experienced symptoms are five times more likely to perceive a higher level of risk. Both the experience of heat-related symptoms and sleeping at uncomfortable temperatures were significantly correlated with elevated health risk perception. This finding aligns with previous research indicating that past experience with heat-related issues tends to heighten awareness and risk perception [11]. Statistically significant associations were identified between the experience of heat-related symptoms and variables such as sex (higher among men), educational attainment (lower levels of education), employment status (unemployed), health status (fair), and pre-existing chronic conditions. These associations facilitate the identification of the groups most sensitive and vulnerable to extreme heat in Puerto Rico.

The majority of the participants (68%) reported experiencing sleep at temperatures deemed uncomfortable. Exposure to nighttime heat was significantly correlated with an increased likelihood of experiencing heat-related symptoms (OR = 1.85; *p* = 0.009). Elevated nighttime temperatures have been linked to heightened health risks and excess mortality, particularly among individuals without access to air conditioning [5,34]. Consequently, addressing nighttime heat exposure, particularly among vulnerable populations, is a crucial strategy for mitigating public health risks.

Among the measures adopted to mitigate heat, approximately half of the participants (45%) reported visiting a public space to cool off during summer, albeit infrequently. This behavior was more frequently employed among young adults (21–34 years) and less frequently among older adults (65 years). Potential limitations in mobility among older adults, coupled with the isolation measures recommended during the 2020 COVID-19 pandemic, may have contributed to the reported infrequency of visits to public spaces for cooling among this demographic. The two main strategies reported to mitigate heat in the home environment were the use of fans and air conditioning (AC). However, the implementation of these strategies faces structural barriers owing to the economic constraints that limit AC usage, deficiencies in the electrical system in Puerto Rico, the frequency and duration of power outages, and the vulnerability associated with tropical cyclones and their effects on the heat and electrical infrastructure. These factors may impair the capacity of Puerto Rico′s residents, particularly the most vulnerable population, to adapt to extreme heat conditions, thereby exacerbating existing inequalities.

This study acknowledges several limitations that should be considered when interpreting the findings. First, the cross-sectional nature of the study inherently limits the ability to establish causal links. Second, there is a possibility of recall bias, especially concerning subjective measures, which was due to the time lapse between the exposure period and data collection. Third, household income data were constrained by a significant amount of missing or incomplete responses. This limitation hindered our capacity to examine the influences of socioeconomic status and poverty on heat-related perceptions and behaviors, which are recognized as determinants of health outcomes in the context of climate-related stressors. However, the inclusion of variables such as educational level and employment status provided indirect insights into the socioeconomic disparities within the sample. Fourth, this study did not consider humidity levels, which significantly influence heat perception and physiological stress in tropical climates, like that of Puerto Rico, potentially affecting the accuracy of heat exposure assessments. Finally, as with all self-reported survey data, the possibility of response and social desirability biases exists, especially concerning behaviors and attitudes related to climate change and health, which may be influenced by awareness or perceived expectations.

## 5. Conclusions

This research reveals critical gaps in heat risk perception among Puerto Rico residents, with only one in four recognizing high levels of future risk despite current exposure concerns. Key vulnerable groups include non-metropolitan residents, men, adults aged 50–64, non-employed individuals, those with fair health and chronic conditions, and people experiencing nighttime heat discomfort. Priority interventions should focus on risk communication to vulnerable groups, emphasizing nighttime heat dangers and promoting protective behaviors. Addressing Puerto Rico′s electrical infrastructure challenges is essential for effective heat mitigation. Solutions should include decentralized energy systems, cooling centers with backup power, and passive thermal infrastructure to reduce electricity dependence and strengthen community resilience against increasing heat-related risks.

## Figures and Tables

**Table 1:** Distribution of the study sample by region, age, and gender comparison of the adult population in Puerto Rico and persons sampled (N = 500).

Category	Persons 21 Years of Age or Older (Puerto Rico) ^1^	Percentage (%)	Persons Sampled ^2^
**By Region**			
Metropolitan	725,277	28.4%	142
Non-metropolitan	1,827,386	71.6%	358
Total (Region)	2,552,663	100%	500
**By Gender and Age Group**			
Men 21 to 34 Years	301,079	11.8%	59
Men 35 to 49 Years	304,859	11.9%	60
Men 50 to 64 Years	302,698	11.9%	59
Men 65 Years or More	278,729	10.9%	55
Women 21 to 34 Years	309,238	12.1%	60
Women 35 to 49 Years	337,954	13.2%	66
Women 50 to 64 Years	356,212	14.0%	70
Women 65 Years or More	361,894	14.2%	71
Total (Gender and Age)	2,552,663	100%	500

^1^ Source: U.S. Census Bureau, Puerto Rico Community Survey 2018 (5-year estimate), ^2^ One adult (21+) was randomly selected from each household for an interview.

**Table 2:** Sociodemographic characteristics of the survey participants (N = 500).

Variable	Category	Frequency (N)	Percentage (%)
Region	Metropolitan	142	28.4%
	Non-metropolitan	358	71.6%
Gender	Female	233	46.6%
	Male	267	53.4%
Age (Years)	21–34	119	23.8%
	35–49	126	25.2%
	50–64	129	25.8%
	65 or More	126	25.2%
Education (N = 498)	Less Than High School	52	10.4%
	High School	141	28.3%
	Assoc. Degree/Certificate	78	15.7%
	Bachelor’s Degree	156	31.3%
	Master’s or Doctoral Degree	71	14.3%
Employment Status (N = 490)	Employed	207	42.2%
	Unemployed	103	21.0%
	Retired	151	30.8%
	Unable to Work	39	8.0%
Work Environment (N = 207)	Interior	131	63.3%
	Exterior	29	14.0%
	Interior/Exterior	47	22.7%
Persons per Household	1	93	18.6%
	2 to 3	281	56.2%
	4 or More	126	25.2%
Health Insurance (N = 495)	Yes	472	95.4%
	No	23	4.6%
Type of Health Insurance (N = 495)	Private	226	45.7%
	Public (Reforma)	155	31.3%
	Medicare	91	18.4%
	No Insurance	23	4.6%

**Table 3:** Distribution of household size categories across age groups of respondents.

	Household Size (Number of Persons)						
Age Group	One (1)	Percentage (%)	Two to Three	Percentage (%)	Four or More	Percentage (%)	Total
21–34 Years	14	12%	63	53%	42	35%	119
35–49 Years	17	13%	65	52%	44	35%	126
50–64 Years	28	22%	75	58%	26	20%	129
65+ Years	34	27%	78	62%	14	11%	126
Total	93		281		126		500

**Table 4:** Health and chronic condition status (N = 500).

Variable	Category	Frequency (N)	Percentage (%)
Health Status (Self-Reported) (N = 499)	Excellent/Good	346	69.3%
	Fair	127	25.5%
	Poor/Very Poor	26	5.2%
Number of Health Conditions (N = 498)	None	147	29.5%
	1	127	25.5%
	2 to 3	133	26.7%
	4 or More	91	18.3%
Type of Health Conditions (N = 498)	Cardiovascular	199	40.0%
	Metabolic	151	30.3%
	Respiratory	116	23.3%
	Neurological and Sleep Disorders	188	37.8%
	Mental Health	38	7.6%
	Other Chronic Conditions	35	7.0%

**Table 5:** Distribution of self-reported concern and perceived risk related to heat and climate threats.

Variable	Category	Frequency (N)	Percentage (%)
Perceived Health Risk (N = 499)	Low	157	31.5%
	Moderate	107	21.4%
	High	235	47.1%
Perceived Personal Likelihood of Heat-Related Illness (5 Years) (N = 479)	Low	277	57.8%
	Moderate	84	17.5%
	High	118	24.6%
Perceived Family-Level Likelihood of Heat-Related Illness (5 Years) (N = 455)	Low	266	58.5%
	Moderate	83	18.2%
	High	106	23.3%
Perceived Community-Level Likelihood of Heat-Related Illness (5 Years) (N = 329)	Low	154	46.8%
	Moderate	80	24.3%
	High	95	28.9%
Climate-Change Concern (N = 497)	Low	41	8.2%
	Moderate	61	12.3%
	High	395	79.5%
High-Temperature Concern (N = 500)	Low	49	9.8%
	Moderate	59	11.8%
	High	392	78.4%
Heatwave Concern (N = 499)	Low	56	11.2%
	Moderate	62	12.4%

**Table 6:** Variables significantly associated with perceived health risk (Chi-squared test, *p*-value < 0.05).

Variable	Perceived Health Risk	Future Personal Risk ^1^	Future Family Risk ^1^	Future Community Risk ^1^
Region	**0.047**	0.090	0.184	0.370
Age	**<0.001**	**0.001**	0.073	0.336
Gender	**<0.001**	**<0.001**	**0.024**	0.183
Educational Attainment	0.142	**0.023**	0.242	0.480
Employment Status	**0.009**	**0.002**	**0.002**	0.222
Occupational Environment	0.293	0.138	**0.040**	0.643
Health Status	**0.015**	**<0.001**	**0.031**	0.132
Number of Health Conditions	**0.001**	**<0.001**	0.074	**0.039**
2 or More Chronic Conditions	**0.031**	**<0.001**	**0.015**	**0.007**
3 or More Chronic Conditions	**0.002**	**<0.001**	**0.005**	**0.029**
4 or More Chronic Conditions	**<0.001**	**<0.001**	0.093	0.313
Experienced Heat-Related Symptoms	**<0.001**	**<0.001**	**<0.001**	**<0.001**
Experienced Heat Discomfort while Sleeping	**0.018**	**0.006**	**<0.001**	**<0.001**
Visit Public Place (to Cool Off)	0.312	0.385	**0.008**	**0.045**

^1^ Perceived risk of heat-related illness (5 years). Only variables with statistically significant associations (*p* < 0.05) according to the Chi-squared test are shown. Values in bold indicate statistical significance.

**Table 7:** Perceived heat exposure and heat-related behaviors.

Variable	Category	Frequency (N)	Percentage (%)
Experienced Heat-Related Symptoms (N = 500)	No	282	56.4%
	Yes	218	43.6%
Frequency of Symptoms (N = 218)	1–2 times	97	44.5%
	2–4 times	74	33.9%
	5 or more	47	21.6%
Visited Public Places to Cool Off (N = 496)	No	275	55.4%
	Yes	221	44.6%
Frequency of Visits (N = 221)	Low	139	62.9%
	High	82	37.1%
Experienced Heat Discomfort while Sleeping (N = 498)	No	159	31.9%
	Yes	339	68.1%
Frequency Heat Discomfort while Sleeping (N = 339)	Low	233	68.7%
	High	106	31.3%
Heat Mitigation Behaviors (N = 497)	AC	319	64.2%
	Fans	418	84.1%
	Window/Door Opening	197	39.6%
	Others	23	4.6%
AC Usage (Hours per Day) (N = 306)	<8 h	59	19.3%
	8–12 h	109	35.6%
	13–24 h	138	45.1%
Uncomfortable Outdoor Temperature (N = 408)	≤85 °F	112	27.5%
	≥86 °F	296	72.5%

**Table 8:** Profile of heat-sensitive and -vulnerable populations in Puerto Rico.

	**Health Risk Perception (Category with the Highest Percentage (%))**				
**Variable**	**High**	**Percentage (%)**	**Low**	**Percentage (%)**	*p*-Value *
Region	Non-metropolitan	48.7	Metropolitan	39.4	**0.047**
Gender	Male	55.8	Female	37.9	**<0.001**
Age (Years)	50–64	63.3	65+	38.9	**<0.001**
Educational Level	<High School	59.6	Graduate Studies	38	0.142
Persons per Household	4 or More	49.6	1 (Living Alone)	40.6	0.310
Employment Status	Unable to Work	75.0	Retired	35.1	**0.009**
Working Environment	Exterior	55.2	Interior	38.2	0.293
Health Status	Regular	58.7	Excellent/Good	35.8	**0.015**
Number of Conditions	4 or More	65.6	None	35.4	**0.001**
Heat-Related Symptoms	Yes	66.8	No	43.3	**<0.001**
Uncomfortable Sleep Temp.	Yes	50.9	No	39.6	**0.018**
Thermal Discomfort	<86 °F	51.8	≥86 °F	27.8	0.856
	**Heat-Related Symptoms (Category with the Highest Percentage (%))**				
**Variable**	**Yes**	**Percentage (%)**	**No**	**Percentage (%)**	*p*-Value *
Region	Non-metropolitan	45.5	Metropolitan	61.3	0.167
Gender	Male	48.3	Female	61.8	**0.023**
Age (Years)	50–64	51.9	65+	61.1	0.149
Educational Level	<High School	59.6	Graduate Studies	66.2	**0.034**
Persons per Household	4 or More	48.4	2 to 3	58.7	0.405
Employment Status	Unable to Work	69.0	Employed	61.4	**0.010**
Working Environment	Interior/Exterior	46.8	Interior	64.9	0.350
Health Status	Regular	67.7	Excellent/Good	66.5	**<0.001**
Number of Conditions	4 or More	67.0	None	70.1	**<0.001**
Uncomfortable Sleep Temp.	Yes	48.7	No	67.3	**<0.001**
Thermal Discomfort	<86 °F	52.7	≥86 °F	53.7	0.249

* Bolded values indicate statistical significances at *p* < 0.05 in chi-squared tests.

**Table 9:** Multinomial logistic regression models for perceived heat-related health risks.

	OR (Adjusted)	95% CI	*p*-Value
**1. Perceived Health Risk Associated with Heat**			
Region			
Metropolitan	1.00		
Non-metropolitan	**1.69**	**(1.00, 2.84)**	**0.047**
Age			
21–34 Years	1.00		
35–49 Years	1.40	(0.73,2.70)	0.319
50–64 Years	**3.59**	**(1.66, 7.77)**	**0.001**
65+ Years	1.29	(0.50, 3.33)	0.596
Gender			
Female	1.00		
Male	**2.00**	**(1.24, 3.23)**	**0.005**
Experienced Heat-Related Symptoms			
No	1.00		
Yes	**4.94**	**(2.93, 8.34)**	**0.000**
(Number of obs. = 484; LR chi^2^ (30) = 126.04; prob. > chi^2^ = <0.001; pseudo R^2^ = 0.1246)			
**2. Perceived Individual Risk of Heat-Related Illness (5 Years)**			
Age			
21–34 Years	1.00		
35–49 Years	1.45	(0.63, 3.38)	0.385
50–64 Years	**2.78**	**(1.25, 6.19)**	**0.012**
65+ Years	0.959	(0.39, 2.34)	0.928
Gender			
Female	1.00		
Male	**3.28**	**(1.81, 5.93)**	**0.000**
Experienced Heat-Related Symptoms			
No	1.00		
Yes	**2.98**	**(1.66, 5.36)**	**0.000**
(Number of obs. = 385; LR chi^2^ (34) = 109.39; prob. > chi^2^ = <0.001; pseudo R^2^ = 0.1433)			
**3. Perceived Family Risk of Heat-Related Illness (5 Years)**			
Gender			
Female	1.00		
Male	**1.82**	**(1.01, 3.27)**	**0.045**
Experienced Heat-Related Symptoms			
No	1.00		
Yes	**3.43**	**(1.91, 6.16)**	**0.000**
(Number of obs. = 352; LR chi^2^ (34) = 77.77; prob. > chi^2^ = <0.001; pseudo R^2^ = 0.1108)			
**4. Perceived Community Risk of Heat-Related Illness (5 Years)**			
Experienced Heat-Related Symptoms			
No	1.00		
Yes	**2.56**	**(1.42, 4.62)**	**0.002**
Slept in Heat Discomfort			
No	1.00		
Yes	**2.53**	**(1.34, 4.79)**	**0.004**
(Number of obs. = 321; LR chi^2^ (26) = 57.01; prob. > chi^2^ = 0.004; pseudo R^2^ = 0.0840)			

All the models used low levels of perceived risk as the reference categories. The odds ratios (ORs) shown represent the likelihood of reporting high levels of perceived risk compared to low levels of perceived risk. The results for the moderate-risk category were excluded from the table to avoid redundancy, as they were fewer and less consistent. Values in bold indicate statistical significance. Only variables with *p* < 0.05 in the adjusted model are shown. Adjusted models included the following covariates: 1: region, age, gender, employment status, health status, number of chronic conditions, experienced heat-related symptoms, and slept in heat discomfort; 2: region, age, gender, educational level, health status, number of chronic conditions, experienced heat-related symptoms, slept in heat discomfort, and thermal discomfort threshold; 3: region, age, gender, employment status, health status, number of chronic conditions, experienced heat-related symptoms, slept in heat discomfort, visited a public place to cool down, and thermal discomfort threshold; 4: region, gender, health status, number of chronic conditions, experienced heat-related symptoms, slept in heat discomfort, and visited a public place to cool down.

**Table 10:** Binary logistic regression models for heat-related symptoms.

Variable	Unadjusted OR	(95% CI)	*p*-Value	Adjusted OR *	(95% CI)	*p*-Value
Gender						
Female	1.00					
Male	1.51	(1.06, 2.16)	0.023	1.37	(0.89, 2.11)	0.150
Education						
Less Than High School	1.00			1.00		
High School	0.53	(0.28, 1.01)	0.055	0.71	(0.32, 1.58)	0.396
Associate Degree or Certificate	0.68	(0.33, 1.38)	0.282	1.07	(0.44, 2.60)	0.878
Bachelor′s Degree	0.45	(0.24, 0.85)	0.014	0.81	(0.34, 1.92)	0.636
Graduate Degree	0.35	(0.16, 0.73)	0.005	0.70	(0.27, 1.86)	0.479
Employment Status						
Employed	1.00			1.00		
Unemployed	1.5	(0.93, 2.41)	0.097	0.89	(0.49, 1.63)	
Retired	1.07	(0.70, 1.65)	0.738	0.87	(0.41, 1.86)	
Unable to Work	3.52	(1.53, 8.13)	0.003	1.15	(0.41, 3.29)	0.788
Health Status						
Excellent/Good	1.00					
Fair	4.15	(2.70, 6.42)	0.000	**3.07**	**(1.79, 5.25)**	**0.000**
Poor/Very Poor	3.17	(1.39, 7.21)	0.006	2.08	(0.73, 5.90)	0.170
Number of Chronic Conditions						
None	1.00					
1 Condition	1.38	(0.83, 2.28)	0.216	1.30	(0.74, 2.33)	0.360
2–3 Conditions	2.30	(1.41, 3.76)	0.001	**1.95**	**(1.07, 3.56)**	**0.029**
4 or More Conditions	4.75	(2.71, 8.35)	0.000	**3.09**	**(1.48, 6.42)**	**0.003**
Health Insurance Type						
Private	1.00					
Public (Reforma)	1.97	(1.31, 2.99)	0.001	1.29	(0.76, 2.21)	0.338
Medicare	1.06	(0.65, 1.75)	0.803			
Slept in Heat Discomfort						
No	1.00					
Yes	1.95	(1.32, 2.89)	0.001	**2.11**	**(1.31, 3.41)**	**0.002**
Use AC						
No	1.00					
Yes	0.62	(0.43, 0.90)	0.012	0.84	(0.51, 1.39)	0.508

Only variables with *p* < 0.05 in unadjusted regressions are included. Adjusted ORs are from a multivariate model including the following covariates: age, gender, employment status, educational level, health status, number of chronic conditions, AC use, slept in heat discomfort, and type of health insurance. Bolded values indicate statistical significance at *p* < 0.05 in adjusted model. * Model statistics: Number of obs. = 458; LR chi^2^ (20) = 87.69; prob. > chi^2^ =< 0.001; pseudo R^2^ = 0.1398.

## Data Availability

The data presented in this study are available on request from the corresponding author due to ethical and privacy restrictions and may require approval from the Institutional Review Board (IRB).

## Appendix A

**Table A1:** Survey variables.

Domain/Variable	Description	Type	Survey Response Options	Analytical Coding
Sociodemographic and Socioeconomic (Social Determinants)				
Geographic region	Region in which the respondent resides, determined by the municipality of residence	Nominal	Municipality of residence (coded as 1–78) and grouped in two regions: Metropolitan (Bayamon, Carolina, Cataño, Guaynabo, San Juan, Toa Baja, and Trujillo Alto) and Non-metropolitan (confirmed by the rest of the municipalities)	(1) Metropolitan (2) Non-metropolitan
Age	Participant’s age in years	Categorical (ordinal)	Exact age recorded; age group selected by enumerator: (1) 21–34, (2) 35–49, (3) 50–64, or (4) 65 or more	(1) 21–34, (2) 35–49, (3) 50–64, or (4) 65 or more
Gender	Gender of the respondent at birth	Nominal	(1) Female, (2) Male, or (3) Other	(1) Female, (2) Male, or (3) Other
Employment Status	Employment Status	Nominal	(1) Employed, (2) Unemployed, (3) Retired, or (4) Unable to Work	(1) Employed, (2) Unemployed, (3) Retired, or (4) Unable to Work
Household Size	The total number of people currently living in the respondent′s household, including self.	Categorical (ordinal)	Any whole number	Recoded as (1) 1; (2) 2 to 3; (3) 4 or more
Household Income	The total annual household income (USD) from all sources, before taxes	Categorical (ordinal)	1 = Less than $10,000 2 = $10,000–$14,999 3 = $15,000–$24,999 4 = $25,000–$34,999 5 = $35,000–$49,999 6 = $50,000–$74,999 7 = $75,000 or more	Same as collected
Educational Attainment	The highest level of education completed	Categorical (ordinal)	1 = Less than high school 2 = High school diploma 3 = Bachelor’s degree 4 = Master’s degree 5 = Doctoral degree 6 = Other (open)	Recoded as (1) less than high school, (2) high school, (3) technical/vocational course or associate degree, (4) bachelor′s degree, or (5) master′s degree or doctoral degree
Health Insurance Coverage	Indicates whether the respondent has health insurance.	Binary (categorical)	1 = Yes, private insurance 2 = Yes, government health plan (Plan VITAL, known as “La Reforma de Salud”) 3 = Yes, Medicare 4 = No health insurance	Recoded: 0 = No (response 4); 1 = Yes (responses 1–3)
Type of Health Insurance	Type of insurance among those with coverage	Categorical (nominal)	1 = Yes, private insurance 2 = Yes, government health plan (Plan VITAL, known as “La Reforma de Salud”) 3 = Yes, Medicare 4 = No health insurance	Recoded: 1 = Private; 2 = Government; 3 = Medicare; response 4 (No) excluded
Health Status and Conditions				
Health Status	Self-rated general health status reported by the respondent	Categorical (ordinal)	1 = Excellent 2 = Good 3 = Fair 4 = Poor 5 = Very Poor	Recoded: 1 = Excellent/Group (responses 1–2); 2 = Fair (response 3); 3 = Poor/Very Poor (responses 4–5)
Health Conditions (15 items) (Descriptive)	Health conditions reported by the respondent across 15 listed health conditions.	Categorical (nominal)	Yes/No	Used descriptively; conditions grouped in broader categories
Number of Health Conditions	The total number of conditions reported across 15 listed health conditions	Continuous (discrete) Categorical (ordinal)	Yes/No	The total count of “yes” responses. Grouped: 0 = None; 1 = 1 condition; 2 = 2–3; 3 = 4 or more
The Minimum Number of Health Conditions	Binary variables derived from the total number of reported health conditions, indicating whether the respondent had 1 or more, 2 or more, 3 or more, or 4 or more conditions.	Binary (categorical)	Derived from the total count of conditions (Yes responses)	Yes = 1 or No = 0 (for each threshold, 1, 2, 3, and 4)
Heat Risk Perception				
Perceived Health Risk	Self-reported level of concern about personal health during hot weather	Categorical (ordinal)	Likert scale from 1 to 5, where 1 = “Not concerned at all” and 5 = “Extremely concerned”	Recoded: 1 = Low (scores 1–2), 2 = Moderate (3), or 3 = High (scores 4–5)
Perceived Likelihood of Personal Illness	Perceived likelihood of experiencing a heat-related illness requiring medical attention within 5 years	Categorical (ordinal)	Likert scale from 1 to 5, where 1 = “Very unlikely” and 5 = “Very likely”	Recoded: 1 = Low (scores 1–2), 2 = Moderate (3), or 3 = High (scores 4–5)
Perceived Likelihood of Family Illness	Perceived likelihood of a family member experiencing a heat-related illness requiring medical attention within 5 years	Categorical (ordinal)	Likert scale from 1 to 5, where 1 = “Very unlikely” and 5 = “Very likely”	Recoded: 1 = Low (scores 1–2), 2 = Moderate (3), or 3 = High (scores 4–5)
Perceived Likelihood of Community Illness	Perceived likelihood of someone in the community experiencing a heat-related illness requiring medical attention within 5 years	Categorical (ordinal)	Likert scale from 1 to 5, where 1 = “Very unlikely” and 5 = “Very likely”	Recoded: 1 = Low (scores 1–2), 2 = Moderate (3), or 3 = High (scores 4–5)
Descriptive Heat Risk Perception				
Primary Reason for Heat Concern	Open-ended question where participants stated their main reason for concern during hot weather	Thematic categories (descriptive)	Open-ended response	Grouped: Direct health effects (responses indicating immediate physical symptoms or direct exposure to heat); indirect health effects (responses indicating secondary consequences or the worsening of existing conditions); other (environmental or other concerns)
Climate-Related Concerns				
Concern about Climate Change	Level of concern about climate change	Categorical (ordinal)	Likert scale from 1 to 5, where 1 = “Not concerned at all” and 5 = “Extremely concerned”	Recoded: 1 = Low (scores 1–2), 2 = Moderate (3), or 3 = High (scores 4–5)
Concern about Hot Temperatures	Level of concern about increasing hot temperatures	Categorical (ordinal)	Likert scale from 1 to 5, where 1 = “Not concerned at all” and 5 = “Extremely concerned”	Recoded: 1 = Low (scores 1–2), 2 = Moderate (3), or 3 = High (scores 4–5)
Concern about Heatwaves	Level of concern about extreme heat events or heatwaves	Categorical (ordinal)	Likert scale from 1 to 5, where 1 = “Not concerned at all” and 5 = “Extremely concerned”	Recoded: 1 = Low (scores 1–2), 2 = Moderate (3), or 3 = High (scores 4–5)
Perceived Heat Exposure				
Threshold Temperature for Outdoor Discomfort	Self-reported temperature (F) for outdoor heat discomfort based on combined responses from a structured and an open-ended question	Continuous (numeric) Binary (categorical)	Open numeric responses in °F or °C	Used as reported for descriptive analysis; values standardized to °F Grouped: 1 = Below 86 °F; 2 = 86 °F or higher
Frequency of Heat-Related Symptoms	The number of times the respondent experienced symptoms believed to be heat-related during summer, such as fainting, rapid heartbeat, hallucinations, confusion, dizziness, or muscle pain	Categorical (ordinal)	Open numeric response	Grouped: 1 = 1–2 times; 2 = 3–4 times; 3 = 5 or more
Experienced Heat-Related Symptoms	Binary indicator of whether the respondent experienced heat-related symptoms during the summer of 2020	Binary (categorical)	Derived from the frequency of heat-related symptoms	0 = No (frequency = 0); 1 = Yes (frequency ≥ 1)
**Heat-Related Behaviors**				
Frequency of Visits to a Public Space to Cool down	The frequency with which the respondent or household members visited public spaces (e.g., malls and restaurants) specifically to cool down during the summer of 2020	Categorical (ordinal)	Five-point scale from 1 to 5, where 1 = “Never” to 5 = “Very frequently”	Recoded: 0 = Never (score 1); 1 = Low (scores 2–3), 2 = High (scores 4–5)
Visited a Public Space to Cool down	Binary indicator of whether respondents visited a public space to cool down	Binary (categorical)	Derived from 5-point scale responses	Recoded: 0 = No (score = 1); 1 = Yes (score ≥ 1)
Frequency: Slept in Uncomfortably Hot Temperatures	Frequency with which the respondent slept in uncomfortably hot temperatures during the summer of 2020	Categorical (ordinal)	Five-point scale: 1 = Never, 2 = Rarely, 3 = Sometimes, 4 = Often, or 5 = Always	Recoded: 0 = Never (score 1); 1 = Low (scores 2–3); 2 = High (scores 4–5)
Slept in Uncomfortably Hot Temperatures	Binary indicator of whether respondents ever slept in uncomfortably hot temperatures	Binary (categorical)	Derived from frequency scale	Recoded: 0 = No (score 1); 1 = Yes (score ≥ 1)
Used AC	Used air conditioning at home to stay cool (predefined survey option: “Used air conditioning”)	Binary (categorical)	Selected/Not Selected	Recoded: 0 = No; 1 = Yes
Used Fans	Used fans at home to stay cool (predefined survey option: “Used ceiling fans or others”)	Binary (categorical)	Selected/Not Selected	Recoded: 0 = No; 1 = Yes
Opened Windows and Doors at Night	Opened windows and doors at night to stay cool (predefined survey option)	Binary (categorical)	Selected/Not Selected	Recoded: 0 = No; 1 = Yes
AC Usage (hours)	Average number of hours per day air conditioning was used during the summer of 2020	Continuous (numeric)	Open numeric response	Grouped: 1 = < 8 h; 2 = 8–12; 3 = 13–24 h
**Descriptive Heat-Related Behaviors**				
Reduced AC Use Due to Finances	Whether financial limitations reduced AC use during the summer of 2020	Categorical (nominal)	Yes/No	0 = No; 1 = Yes
Unable to Pay Electricity Bill	Whether the household was unable to pay the electricity bill during the summer of 2020	Categorical (nominal)	Yes/No	0 = No; 1 = Yes
Alternative Energy or Generator	Whether the household has a generator or alternative energy system	Categorical (nominal)	1 = Yes, has an electric or gas generator 2 = Yes, has an alternative energy system 3 = Does not have	Used as reported for descriptive analysis
